# National Health Survey 2019: medication obtainment through the Brazilian Popular Pharmacy Program by adults being treated for hypertension and diabetes

**DOI:** 10.1590/SS2237-9622202200004.especial

**Published:** 2022-07-06

**Authors:** Karen Sarmento Costa, Noemia Urruth Leão Tavares, Vera Lúcia Tierling, Veronica Batista Gomes Leitão, Sheila Rizzato Stopa, Deborah Carvalho Malta

**Affiliations:** 1 Universidade Estadual de Campinas, Departamento de Saúde Coletiva, Campinas, SP, Brazil; 2 Universidade de Brasília, Faculdade de Ciências da Saúde, Brasília, DF, Brazil; 3 Ministério da Saúde, Secretaria de Vigilância em Saúde, Brasília, DF, Brazil; 4 Universidade Federal de Minas Gerais, Escola de Enfermagem, Belo Horizonte, MG, Brazil

**Keywords:** Descriptive Epidemiology, National Policy on Pharmaceutical Assistance, Access to Essential Medicines and Health Technologies, Non-communicable Diseases

## Abstract

**Objective:**

To describe the proportion of adults with hypertension and diabetes who obtained medication through the Brazilian Popular Pharmacy Program (*Programa Farmácia Popular*).

**Method:**

Population-based descriptive study, using data from the 2019 Brazilian National Health Survey. The proportion of individuals who obtained at least one type of medication for hypertension and diabetes in the Program was analysed according to socioeconomic and demographic characteristics, by regions and federative units.

**Results:**

The proportion of individuals who obtained medication for hypertension was 45.1% (95%CI 43.7;46.5), and, for diabetes, 51.5% (95%CI 49.5;53.6). Respectively for both conditions, medication obtainment was higher in the South region (54.3%; 95%CI 51.3;57.2 and 59.1%; 95%CI 54.6;63.7) and lower in the higher strata level of education (30.9%; 95%CI 27.7;34.2 and 40.7%; 95%CI 35.1;46.3) and income (24.0%; 95%CI 19.7;28.2 and 28.9%; 95%CI 22.1;35.7).

**Conclusion:**

Regional and socioeconomic inequalities were identified in obtaining medication for hypertension and diabetes through the Program.

**Study contributions**

**Main results**

The proportion of individuals who obtained at least one medication through the Brazilian Popular Pharmacy Program (PFPB) was 45.1% (95%CI 43.7;46.5) for hypertension treatment and 51.5% (95%CI 49.5;53.6) for diabetes, with differences according to the level of education and income.

**Implications for services**

The magnitude of medication obtainment for hypertension and diabetes treatment through the PFPB reinforces the importance of this complementary strategy of access as one of the main sources for obtaining medication for the treatment of these chronic diseases.

**Perspectives**

It is hoped that strategies such as this one be permanent in Brazil, in order to allow the monitoring of medication obtainment by people being treated for hypertension and diabetes, under the perspective of ensuring pharmacotherapy as part of the health care process.

## Introduction

Non-communicable chronic diseases (NCDs) constitute a challenge for public health worldwide, being responsible for the main causes of morbidity and mortality,^1^ which can generate high expenditure for the public health system, as a result of early retirement and absenteeism.^2^

In 2019, those diseases were responsible for approximately 33.2 million deaths worldwide, an increase of 28% in relation to 2000. Globally, cardiovascular diseases accounted for 17.9 million deaths in 2019, and diabetes for 2.0 million in the same year.^1^ According to the National Health Survey-NHS (*Pesquisa Nacional de Saúde* - PNS), in 2013, in Brazil, 45.1% of the population (over 66 million people) reported having at least one non-communicable chronic disease.^3^

As a consequence of the magnitude of NCDs, the World Health Organization (WHO) has established prevention and control priorities for these diseases as of the year 2000.^4^ Since then, WHO has been updating guidelines and strategies, such as the WHO Global NCD Action Plan 2013-2020 and, more recently, this matter was included in the 2030 Agenda for Sustainable Development Goals (SDGs).^5^ ^,^ ^6^

In light of this, Brazil included, in the 2011-2022 Strategic Action Plan to Tackle Non-communicable Diseases in Brazil, among other measures, the expansion of access to free medication for hypertension and diabetes.^7^ Currently, the free access to such medications is ensured by two provision sources: public pharmacies, mainly those belonging to the municipal health care structure, and drugstores affiliated to the Brazilian Popular Pharmacy Program (*Programa Farmácia Popular do Brasil* - PFPB).^8^

The PFPB was created by the Federal Government in 2004, and its main purpose is to complement other actions of the Brazilian National Health System (*Sistema Único de Saúde* - SUS) regarding access to medication by implementing a copayment modality. The PFPB was implemented along the years through three different models: publicly owned pharmacies, terminated in 2017; partnership with private retail drugstores starting in 2006; and, in 2011, exemption from copayment for hypertension, diabetes and antiasthma medication in all the drugstores under the PFPB.^9^

Almost two decades after the implementation of the PFPB, studies, although scarce, have shown the magnitude of its contribution to expand access to medication for the treatment of NCDs in the country,^8^ ^,^ ^10^ ^,^ ^11^ ^,^ ^12^ ^,^ ^13^ as well as regarding its effect on reducing hospitalization and deaths due to hypertension and diabetes.^14^ However, there are differences, mainly regional, in terms of the PFPB’s coverage and, consequently, users’ access.^9^ ^,^ ^15^

In this context, using data from the 2019 PNS, the present study seeks to contribute to the production of evidence about the PFPB. The main objective was to describe the proportion of adults with hypertension and diabetes who obtained medication through the program.

## Methods

This was a population-based descriptive study, which analyses data from the PNS carried out in 2019 by the Brazilian Institute of Geography and Statistics (*Instituto Brasileiro de Geografia e Estatística* - IBGE) in partnership with the Ministry of Health.

Regarding the 2019 PNS, the target population consisted of individuals aged ≥ 15, who resided in permanent private households in Brazil. It was possible to estimate data by region, Federative Units, capitals and metropolitan regions.^16^ The PNS data collection was carried out between August 2019 and March 2020, and the survey sampling plan was published by IBGE.^17^

For the present analysis, the 2019 PNS public domain microdata, available on the IBGE website, were accessed. The variables of interest were selected according to the dictionary of variables available on the IBGE website, and extracted following the technical guidelines.^16^ ^,^ ^17^

All of the interviewed individuals aged ≥ 18 who reported a medical diagnosis of hypertension and diabetes, indication and use of medication for these conditions in the two weeks prior to the interview were included. Obtainment of at least one type of medication through the PFPB was considered for those who responded "yes" to the following questions:

- *Was any of the drugs for hypertension obtained through the “Aqui tem farmácia popular” (There is a popular pharmacy here program)?*
- *Was any of the oral drugs for diabetes obtained through the “There is a popular pharmacy here”?*
- *Was insulin obtained through the “There is a popular pharmacy here”?*

The percentages of people with hypertension and diabetes who obtained at least one medication for the treatment of these diseases in relation to the total of people who reported the diagnostics, indication and use of medication were estimated. The percentages are presented according to socioeconomic and demographic variables and their distribution in the Brazilian regions and Federative Units.

Data analysis in the present article was performed based on a sample stratified by: sex (male; female); age groups (in years: 18 to 29; 30 to 59; 60 to 64; 65 to 74; and 75 or over); declared race/skin color (White; Black; Brown), being the Yellow color or indigenous ethnic groups included only in the total, due to the small number of observations and high coefficient of variation; educational level (no schooling and incomplete primary education; complete primary education and incomplete secondary education; complete secondary education and incomplete higher education; complete higher education); and monthly income [no income or up to 1/4 of the minimum wage (MW); more than 1/4 of the MW to 1/2 MW; more than 1/2 to 1 MW; more than 1 to 2 MWs; more than 2 to 3 MWs; more than 3 to 5 MWs; more than 5 MWs].

Variables with a variation coefficient below 30% were described. Relative frequencies and respective 95% confidence intervals (95%CI) were presented, considering the complex sampling plan and sample weighting using version 9.0 of the SAS software. Frequencies whose confidence intervals did not overlap when comparing the categories of variables of interest were considered different.

The 2019 PNS was sent to the National Research Ethics Committee/National Health Council and approved under Opinion No. 3.529.376, issued on August 23, 2019.

## Results

In 2019, in Brazil, the proportion of individuals ≥ 18 who reported medical diagnosis of hypertension was 23.9% (95%CI 23.5;24.4), which corresponds to more than 38 million people, and, for diabetes, the percentage was 7.7% (95%CI 7.4;8.0), which represents over 12 million people (Table 1).

**Table 1:** Prevalence of individuals aged ≥ 18 who reported medical diagnosis of hypertension and diabetes, and the proportion of diagnosed individuals who obtained at least one medication through the Popular Pharmacy Program, according to region, 2019 National Health Survey, Brazil

Regions	Hypertension				Diabetes			
	Diagnosis		Obtainment through PFPB^a^		Diagnosis		Obtainment through PFPB^a^	
	%	95%CI^b^	%	95%CI^b^	%	95%CI^b^	%	95%CI^b^
Brazil	23.9	23.5;24.4	45.1	43.7;46.5	7.7	7.4;8.0	51.5	49.5;53.6
North	16.8	16.0;17.6	38.0	34.6;41.4	5.5	5.0;6.0	35.6	30.5;40.7
Northeast	23.1	22.5;23.7	34.8	32.9;36.7	7.2	6.8;7.6	39.5	36.4;42.6
Southeast	25.9	25.0;26.8	48.0	45.6;50.5	8.5	8.0;9.1	56.4	52.8;60.1
South	24.5	23.5;25.5	54.3	51.3;57.2	7.9	7.3;8.5	59.1	54.6;63.7
Midwest	21.9	20.9;23.0	47.7	44.3;51.1	7.2	6.5;7.8	56.4	51.7;61.0

a) PFPB: Brazilian Popular Pharmacy Program; b) 95%CI: 95% confidence interval.

Of the total number of people who reported the diagnosis of hypertension, 86.9% (IC95% 86.2;87.7) declared the use of all medications for your treatment, with the highest percentages observed among females (89.6%; 95%CI 88.6;90.6) when compared to males (83.6%; 95%CI 81.8;84.5). For diabetes, the use of some type of medication was reported by 88.8% (95%CI 87.5;90.2), with no sex differences.

For hypertension, the equivalent of more than 15 million Brazilians who reported diagnosis, indication and use of medication, obtained at least one type of medication through the PFPB (45.1%; 95%CI 43.7;46.5). The highest proportion was found in the South region (54.3%; 95%CI 51.3;57.2), with a difference when compared to the other regions. The smallest proportion was observed in the Northeast region (34.8%; 95%CI 32.9;36.7) (Table 1).

For diabetes, about 5.8 million people reported having obtained some type of medication through the PFPB (51.5%; 95%CI 49.5;53.6). The highest proportion was found in the South region (59.1%; 95%CI 54.6;63.7), with a difference in relation to the North (35.6%; 95%CI 30.5;40.7) and Northeast regions (39.5%; 95%CI 36.4;42.6), Table 1.

**Figure 1:**
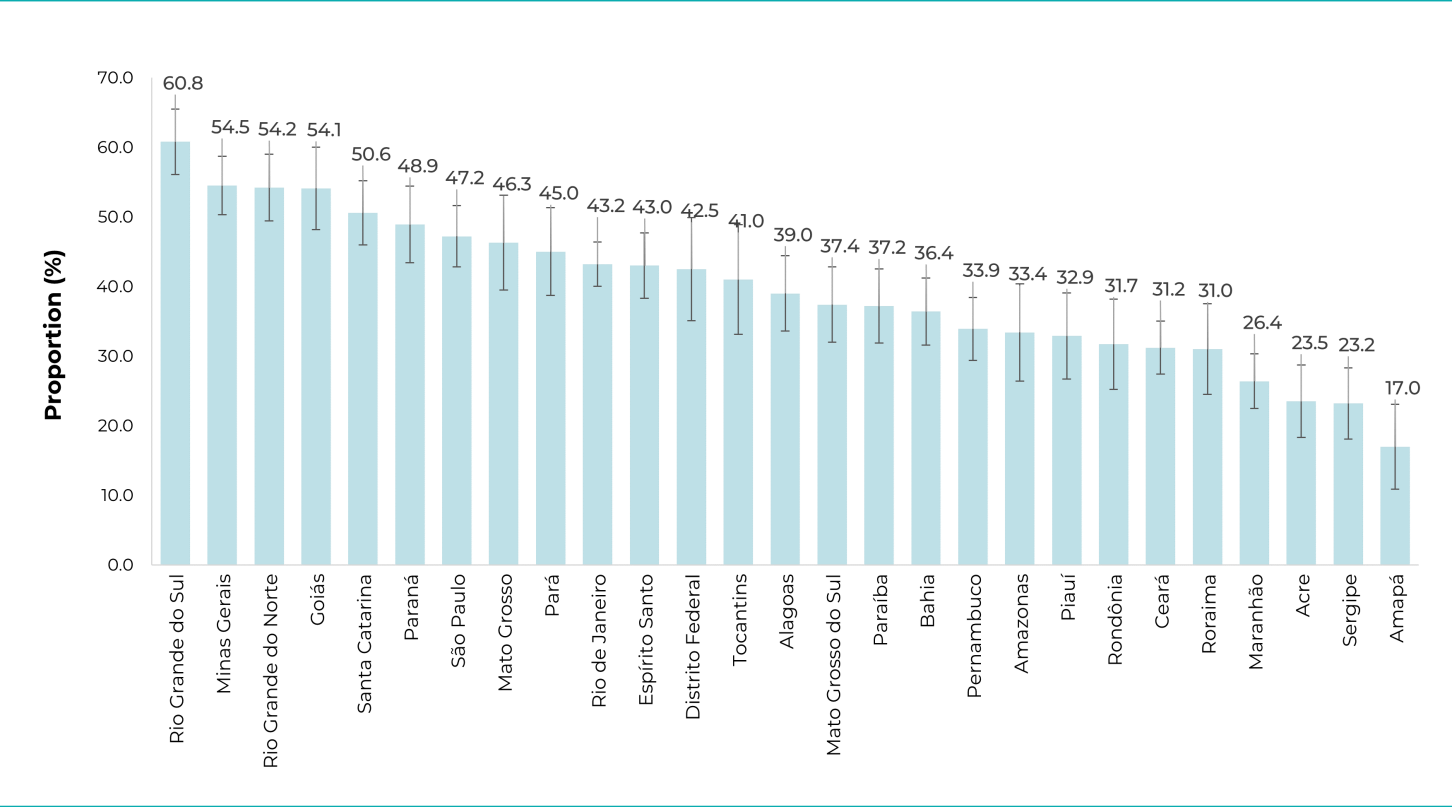
Proportion of individuals aged ≥ 18 with hypertension who reported obtaining antihypertensive medication through the Brazilian Popular Pharmacy Program, by Federative Unit, 2019 National Health Survey, Brazil

Regarding the Federative Units, the highest proportions of antihypertensive medication obtainment through the PFPB were identified in Rio Grande do Sul, Minas Gerais, Rio Grande do Norte, and Goiás, and the smallest proportions in Amapá, Acre, and Sergipe (Figure 1).

**Figure 2:**
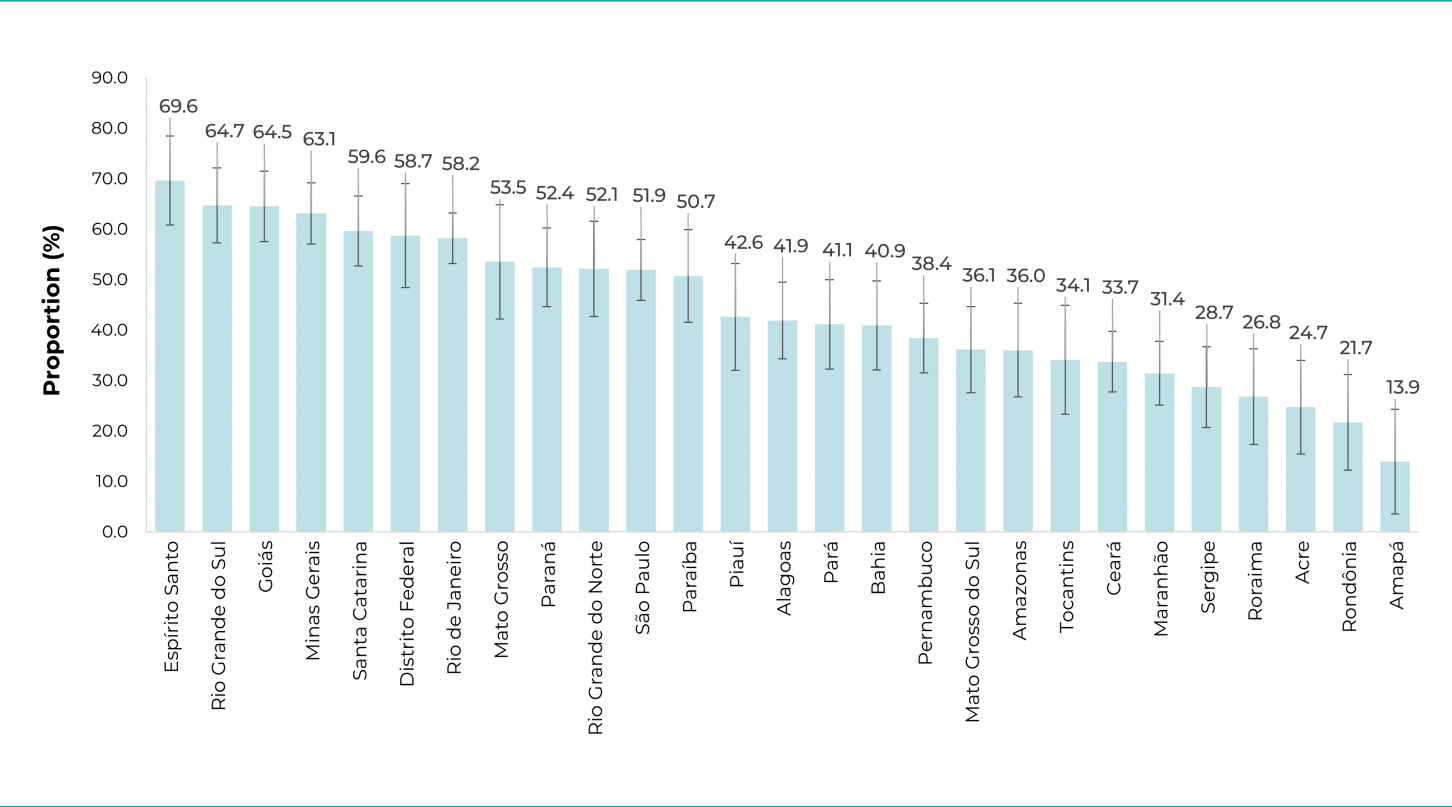
Proportion of individuals aged ≥ 18 with diabetes who reported obtaining antidiabetic medication through the Brazilian Popular Pharmacy Program, by Federative Unit, 2019 National Health Survey, Brazil

With respect to oral drugs and/or insulin for the treatment of diabetes, the highest proportions of medication obtainment through the PFPB were identified in Espírito Santo, Rio Grande do Sul, and Goiás. The lowest percentages, on the other hand, were found in Amapá, Rondônia, and Acre (Figure 2).

For hypertension, differences in medication obtainment through the PFPB were found according to socioeconomic and demographic variables. The percentage for individuals aged 18 through 29 was lower (21.1%; 95%CI 12.7;29.5), when compared to the other age groups analyzed. There was also a decrease in medication obtainment among individuals aged 75 or over (41.5%; 95%CI 38.7;44.3) compared to those aged 60 through 64 (48.3%; 95%CI 45.4;51.3). The proportion of medicines obtained through the PFPB was lower in the strata with higher levels of education and higher income. Differences were detected in the following levels of education: complete secondary education and incomplete higher education (43.0%; 95%CI 40.1;45.9) and complete higher education (30.9%; 95%CI 27.7;34.2) in relation to the other levels. As for income, for those with an income of more than 5 MWs (24.0%; 95%CI 19.7;28.2) the proportion of medication obtainment was lower when compared to the other strata (Table 2).

**Table 2:** Proportion of individuals aged ≥ 18 who reported medical diagnosis of hypertension and obtained at least one type of medication for hypertension through the Popular Pharmacy Program, by socioeconomic and demographic variables, according to region, 2019 National Health Survey, Brazil

Socioeconomic and demographic variables	Brazil		North		Northeast		Southeast		South		Midwest	
	%	95%CI^a^	%	95%CI^a^	%	95%CI^a^	%	95%CI^a^	%	95%CI^a^	%	95%CI^a^
**Sex**												
Male	43.7	41.7;45.7	36.5	31.8;41.3	32.3	29.5;35.0	47.5	44.1;51.0	51.4	47.4;55.4	43.3	37.9;48.7
Female	46.0	44.3;47.7	38.8	34.6;43.0	36.3	34.0;38.5	48.3	45.3;51.4	56.2	52.3;60.1	51.0	47.0;54.9
**Age (in years)**												
18 to 29	21.1	12.7;29.5	37.9	8.7;67.0	19.0	7.5;30.6	16.0	4.3;27.7	49.4	17.5;81.3	3.7	0.0;11.3
30 to 59	45.7	43.4;47.9	40.4	35.8;45.1	35.7	33.1;38.3	49.1	44.9;53.3	51.3	47.0;55.6	49.9	45.0;54.8
60 to 64	48.3	45.4;51.3	32.5	24.8;40.2	35.3	30.5;40.1	55.0	49.9;60.1	55.2	49.3;61.2	44.3	37.2;51.4
65 to 74	45.6	43.3;48.0	36.0	30.3;41.7	32.7	29.0;36.4	48.5	44.5;52.5	59.0	54.5;63.4	50.8	45.2;56.4
75 or over	41.5	38.7;44.3	38.4	31.3;45.6	36.0	31.3;40.7	40.1	35.3;44.9	54.2	47.6;60.8	45.1	37.4;52.7
**Educational level**												
No schooling or incomplete primary education	47.9	46.2;49.6	40.2	36.1;44.3	36.4	34.0;38.9	52.7	49.5;55.9	59.2	55.2;63.2	50.3	45.2;55.4
Complete primary education and incomplete secondary education	50.4	46.7;54.1	47.7	37.5;57.9	36.5	30.5;42.5	52.6	46.5;58.6	57.8	51.6;64.0	53.7	44.6;62.8
Complete secondary education and incomplete higher education	43.0	40.1;45.9	37.0	30.1;43.9	32.6	28.7;36.6	45.4	40.6;50.1	51.1	45.2;57.0	42.6	36.3;49.0
Complete higher education	30.9	27.7;34.2	19.8	12.9;26.6	25.1	20.2;30.1	32.3	27.0;37.6	32.2	25.6;38.9	39.7	32.5;46.9
**Race/skin color**												
White	44.8	42.9;46.7	30.7	24.6;36.8	34.8	31.0;38.5	43.6	40.5;46.7	54.7	51.7;57.7	43.6	38.2;49.0
Black	46.3	42.9;49.7	51.0	44.3;57.7	33.4	28.8;38.0	51.8	46.2;57.3	55.9	45.5;66.2	45.4	36.5;54.4
Brown	44.8	42.9;46.8	37.9	33.7;42.0	34.9	32.6;37.3	52.5	48.7;56.4	52.7	45.1;60.2	51.5	47.0;56.1
**Income (MW)^a^**												
No income to 1/4	44.0	39.2;48.8	39.0	29.7;48.4	36.4	30.7;42.2	53.6	42.9;64.3	47.6	31.6;63.6	56.0	38.2;73.9
1/4 to 1/2	43.7	40.1;47.3	44.0	35.6;52.4	34.3	29.9;38.7	49.5	41.2;57.8	65.8	55.9;75.8	61.6	50.9;72.4
1/2 to 1	48.8	46.3;51.3	43.6	37.8;49.3	37.7	34.7;40.7	54.0	49.3;58.8	59.4	53.7;65.2	50.8	44.6;57.0
1 to 2	48.5	46.3;50.7	33.1	27.7;38.6	34.5	30.5;38.4	51.6	48.2;55.1	56.2	52.0;60.4	48.3	42.8;53.8
2 to 3	42.3	38.0;46.6	29.6	21.1;38.1	34.5	26.5;42.6	39.9	32.8;47.0	54.8	48.1;61.6	44.0	35.2;52.8
3 to 5	36.4	32.2;40.5	27.6	14.7;40.5	21.4	15.3;27.4	38.7	32.7;44.8	41.5	33.2;49.7	34.8	24.6;45.0
Over 5	24.0	19.7;28.2	11.7	5.3;18.2	13.6	8.4;18.8	25.3	18.4;32.1	29.5	21.6;37.4	24.8	17.4;32.3
**Total**	45.1	43.7;46.5	38.0	34.6;41.4	34.8	32.9;36.7	48.0	45.6;50.5	54.3	51.3;57.2	47.7	44.3;51.1

a) 95%CI: 95% confidence interval; b) MW: Minimum monthly wage.

The analysis by region, following what was observed for the country, showed that the Southeast region had the lowest proportion of medication obtainment in the 60 to 64 age group (55.0%; 95%CI 49.9;60.1) when compared to elderly people aged 75 or over (40.1%; 95%CI 35.3;44.9).

In terms of educational level, lower percentages of medication obtainment for the treatment of hypertension were observed in individuals with complete higher education in the North (19.8%; 95%CI 12.9;26.6), South (32.2%; 95%CI 25.6;38.9), and Southeast (32.3%; 95%CI 27.0;37.6) when compared to other levels of education. Regarding income, the proportion of individuals with an income of more than 5 MWs who obtained medication for hypertension was also lower in the North region (11.7%; 95%CI 5.3;18.2) compared to those with an income of 3 MWs or less.

The analysis of the proportions of antihypertensive drug obtainment according to self-reported race/skin color did not show differences for the total population except for the North region, where it was higher among those of Black race/skin color (51.0 %; 95%CI 44.3;57.7) when compared with those of white race/skin color (30.7%; 95%CI 24.6;36.8), as shown in Table 2.

Regarding the obtainment of at least one type of medication for the treatment of diabetes through the PFPB, considering the total population of the country, a lower proportion was observed for younger individuals aged 18 to 29 (36.5%; 95%CI 19.4;53.6), but with no differences in relation to the other strata. Just as for hypertension, a lower proportion of medication obtainment for diabetes through the PFPB was observed for individuals aged 75 or over (43.3%; 95%CI 38.7;47.8) compared to those aged 60 to 64 (56.1%; 95%CI 50.9;61.4), and 65 to 74 (51.9%; 95%CI 48.1;55.7). With reference to the level of education and income, lower proportions were revealed for individuals with complete higher education (40.7%; 95%CI 35.1;46.3) and with an income greater than 5 MWs (28.9%; 95%CI 22.1;35.7) when compared to the other levels analyzed (Table 3).

**Table 3:** Proportion of individuals aged ≥ 18 who reported medical diagnosis of hypertension and obtained at least one medication through the Popular Pharmacy Program, by socioeconomic and demographic variables, according to region, 2019 National Health Survey, Brazil

Socioeconomic and demographic variables	Brazil		North		Northeast		Southeast		South		Midwest	
	%	95%CI^a^	%	95%CI^a^	%	95%CI^a^	%	95%CI^a^	%	95%CI^a^	%	95%CI^a^
**Sex**												
Male	49.0	45.9;52.1	35.1	27.2;43.0	35.7	31.1;40.3	54.0	48.7;59.3	57.0	50.2;63.9	49.0	41.4;56.5
Male	53.4	50.6;56.2	36.0	29.8;42.2	41.9	37.9;45.9	58.4	53.3;63.4	60.7	54.9;66.5	61.3	55.6;66.9
**Age (in years)**												
18 to 29	36.5	19.4;53.6	27.4	0.0;56.9	34.1	10.0;58.2	40.0	4.2;75.9	55.9	12.0;99.9	18.9	0.0;43.5
30 to 59	53.2	49.7;56.8	37.0	28.7;45.3	39.9	35.3;44.5	59.5	53.2;65.7	60.0	52.2;67.8	54.4	46.6;62.3
60 to 64	56.1	50.9;61.4	34.3	22.3;46.3	43.3	35.4;51.1	61.9	53.1;70.7	62.0	51.2;72.7	59.7	49.2;70.3
65 to 74	51.9	48.1;55.7	35.0	26.4;43.7	40.5	35.1;45.9	56.2	49.2;63.2	58.7	51.5;65.9	61.3	52.3;70.2
Over 75	43.3	38.7;47.8	35.3	22.8;47.8	34.3	25.6;43.0	44.5	37.4;51.7	55.0	43.8;66.1	52.3	39.9;64.7
**Educational level**												
No schooling or incomplete primary education	52.3	49.5;55.0	36.0	29.3;42.6	37.6	33.8;41.5	59.3	54.2;64.3	61.8	56.3;67.4	58.4	52.2;64.6
Complete primary education and incomplete secondary education	59.8	54.2;65.4	53.3	39.1;67.5	49.8	40.2;59.5	63.6	54.8;72.4	59.9	47.1;72.7	61.9	48.9;74.8
Complete secondary education and incomplete higher education	50.1	45.4;54.8	35.5	22.5;48.5	43.4	36.4;50.3	52.6	45.0;60.1	54.3	41.7;66.9	53.9	42.9;64.9
Complete higher education	40.7	35.1;46.3	12.0	4.8;19.3	35.7	26.1;45.3	43.1	34.3;51.8	48.5	34.4;62.6	46.7	32.5;60.9
**Race/skin color**												
White	53.6	50.5;56.6	43.0	32.2;53.9	38.5	32.5;44.6	54.0	49.2;58.9	61.4	56.5;66.3	56.2	48.3;64.1
Black	53.8	48.0;59.6	44.6	30.5;58.7	38.2	31.0;45.5	63.5	54.3;72.8	56.0	38.9;73.2	54.1	38.4;69.9
Brown	49.3	46.1;52.5	32.3	26.1;38.4	39.7	35.6;43.7	59.3	52.8;65.7	53.8	42.3;65.2	57.1	50.2;64.0
**Income (MW)^a^**												
No income to 1/4	58.9	51.1;66.8	45.9	30.0;61.8	41.0	31.7;50.4	78.5	66.6;90.5	70.9	49.4;92.3	76.0	55.2;96.8
1/4 to 1/2	51.1	45.6;56.6	34.8	23.2;46.5	40.0	33.2;46.8	65.3	54.0;76.5	62.5	43.6;81.4	56.4	42.0;70.9
1/2 to 1	53.6	50.0;57.3	38.3	29.4;47.2	39.4	34.3;44.5	62.8	56.1;69.6	61.7	52.8;70.5	57.8	48.9;66.8
1 to 2	52.8	48.6;56.9	33.0	22.0;43.9	40.9	34.7;47.0	54.4	47.6;61.3	61.9	54.8;69.0	57.3	47.7;66.9
2 to 3	54.2	46.6;61.8	37.0	20.6;53.4	42.5	29.8;55.1	55.9	43.8;68.0	55.3	41.7;69.0	69.1	56.9;81.2
3 to 5	44.5	37.2;51.7	34.4	17.6;51.1	36.0	23.5;48.6	46.3	35.4;57.2	46.5	30.0;63.0	47.4	33.9;60.9
Over 5	28.9	22.1;35.7	9.0	0.9;17.0	20.3	3.0;37.5	27.8	17.9;37.7	40.2	22.6;57.8	35.3	22.1;48.5
**Total**	51.5	49.5;53.6	35.6	30.5;40.7	39.5	36.4;42.6	56.4	52.8;60.1	59.1	54.6;63.7	56.4	51.7;61.0

a) 95%CI: 95% confidence interval; b) MW: Minimum monthly wage.

In terms of the regions of the country, in the North region there was a lower proportion of medication obtainment for the treatment of diabetes by individuals with complete higher education, compared to other levels of education (12.0%; 95%CI 4.8;19.3), as well as by individuals with an income of more than 5 MWs (9.0%; 95%CI 0.9;17.0), with no differences for the other regions and variables analyzed (Table 3).

Lastly, it was also observed that the obtainment of medication for the treatment of hypertension and diabetes through the PFPB was higher for females, when compared to males, in all major regions, as well as for Brazil, however, with inclusive 95%CI (Tables 2 and 3).

## Discussion

The study revealed that about one fourth of the Brazilian adult population reported having hypertension, and 7.7% diabetes. About half of all the people with diagnosis, prescription and who had been taking medication for the treatment of hypertension and diabetes in Brazil obtained the respective medication through the PFPB in 2019, even though there are regional and socioeconomic inequalities. Lower proportions of medication obtainment were observed for hypertension treatment in the Northeast region, and for diabetes in the North region. However, the proportion of medication obtainment for individuals in the lower levels of education and income strata was higher. Therefore, the relevance of this complementary strategy for the most vulnerable segments of the Brazilian population, in terms of providing access to medication for the treatment of these NCDs is highlighted.

In the past years, several public health programs and strategies worldwide have aimed to control and prevent NCDs,^18^ which reinforces the importance of identifying and monitoring the indicators of prevalence of such diseases and the use of medication.

In Brazil, an increased prevalence of individuals with hypertension (from 21.4% in 2013 to 23.9% in 2019) and diabetes (from 6.2% in 2013 to 7.7% in 2019) has been observed, which can be due, to a large extent, to the obesity epidemic and more access to diagnostic tests,^19^ as well as the ageing of the population, owing to the increased life expectation.^20^

Among the findings, it should be noted that the vast majority of individuals with a medical diagnosis of hypertension or diabetes in Brazil were taking medication, with an increase in these proportions between 2013 and 2019. For hypertension, in 2013, the proportion of medication use was 81.4% and 86.9% in 2019. For diabetes, this proportion was 80.2% in 2013 and 88.8% in 2019.^12^

In 2019, it was noted that over 15 million Brazilians with medically indicated treatment obtained at least one type of medicine for hypertension through the PFPB, a significant growth in relation to what was observed in the 2013 PNS (35.9%).^10^ It is worth highlighting the limitation of the comparison, because in 2013 the estimates for obtaining medication did not consider the medical indication for treatment, since there was no such question in the questionnaire, which could lead to underestimation. This growth also stands out in the analysis of the data collected by the Chronic Disease Risk and Protective Factors Telephone Surveillance Survey (VIGITEL) carried out in the Brazilian capitals, in which an increase in medication obtainment through the PFPB was observed: from 16.1% in 2011 to 29.9% in 2017.^8^

Concerning the importance of the PFPB for access to medication, it is worth highlighting that the increase indicates a change in the medication obtainment pattern, seeing that migration from the use of public pharmacies to the PFPB has been observed in Brazilian capitals since the implementation of gratuity for antihypertensives in 2011.^8^ This result should be analyzed taking the federal expenditures invested in the PFPB into consideration, which, corrected to values of December 31, 2014, showed an average growth of 88% between 2006 and 2014, against the 2% in the volume of transfer of resources to municipalities for the procurement of medicines in the Pharmaceutical Assistance Basic Component in the same period.^21^

In the Brazilian scenario, it is worth noting the severe economic crisis, with the implementation of austerity policies in 2016, such as the reduction in public investment for 20 years in areas such as health and social policies, following the Constitutional Amendment No. 95.^22^ ^-^ ^24^ Leitão and collaborators pointed out that since 2015 there has been a decrease in access to medication in public pharmacies, with people turning to private retail drugstores affiliated with the PFPB.^8^ Therefore, in 2016 and especially in 2017, the observed decrease is possibly due to the austerity policies and restriction of municipal resources resulting from the drop of federal funds transfer.^23^ Such measures reinforce the importance of the PFPB as a complementary strategy to access to free medication in public pharmacies of the public health system.

As for the diabetic population, around 5.8 million people reported having obtained some type of prescribed medication (oral and/or insulin) through the PFPB in 2019, a decrease in relation to 2013: 57.4% or around 4.2 million people.^10^

VIGITEL’s data on oral antidiabetic drug obtainment in the Brazilian capitals between 2012 and 2018 pointed to an increase in obtainment through the PFPB. Nevertheless, the pharmacies in the public health units remained as the main source for obtaining those medications when compared to the pharmacies affiliated to the PFPB.^13^ In addition to oral antidiabetic drugs, the Brazilian National Health System public health units provide insulin and other supplies for glycemic control, which can justify the results found.

Regarding socioeconomic and demographic variables, in 2019 a decrease in medication obtainment for hypertension and diabetes through the PFPB was observed among individuals aged 75 or over, when compared to those in the 60 to 64 age group. This result might be related to a greater need for integrated care in the health system, due to health conditions and more complex treatments with medication not supplied by the PFPB.

In the higher level strata of education (complete higher education) and income (over 5 MWs), both for hypertension and diabetes, lower proportions of medication obtainment through the PFPB were observed. This fact can be an indication that the PFPB, via the complementary strategy of free distribution of medication, has allowed the expansion of access, especially by the most economically disadvantaged social classes.

On the other hand, it is important to draw attention to the regional differences observed in medication obtainment through the drugstores affiliated to the PFPB, with a higher rate in the South region when compared to the Northeast or the North, in relation to both aspects analyzed. This result can indicate inequalities in the utilization of the health services in the Brazilian regions. Studies have shown that, despite the substantial increase in the number of drugstores under the PFPB and its coverage, disparities among the regions remain.^15^ In the states in the South and Southeast regions, which are more economically favored, greater coverage by the PFPB has been identified in contrast with the poorer localities, which can impact the access to medication.^9^

In addition to the classical limitation of cross-sectional studies in determining causality, it is worth highlighting the limitation of the lack of identification of the drugs used by the individuals, which prevents a more in-depth assessment of the gaps in obtaining treatment for the conditions analyzed. However, we emphasize that the changes in the PNS questionnaire from 2013 to 2019 qualified the assessment on the use of medication. In the 2019 edition, a new question was included, making it possible to consider, in the estimates of medication use and obtainment, only individuals with medically indicated treatment. Also, with respect to treatment for diabetes, data related to oral medicines and insulin were collected separately, while in 2013 the questions treated such medications indistinctly.

Another important change was related to the options of sources for obtaining medication. In the 2013 survey, the first option for the source for medication obtainment was “health insurance”, which may have led to underestimation of medication obtainment through SUS and the PFPB by presenting an option of access to medication for hypertension and diabetes which, in practice, for the supply of outpatient medications, is restricted to very few plans and covered by reimbursement.

Lastly, we emphasize that data gathered through national surveys have demonstrated the magnitude of medication obtainment for the treatment of hypertension and diabetes through the PFPB, reinforcing this complementary access strategy as one of the main sources for obtaining medication for the treatment of these chronic conditions in the country, despite the still evident regional inequalities that must be observed in the improvement of the PFPB by SUS managers.
